# Experiences on H.M.S. "Aboukir"

**Published:** 1914-10-17

**Authors:** 


### Experiences on H.M.S. "Aboukir."

### THE ACTION OF TORPEDOES AND SUBMARINES.

We have received the following account of the experiences of Mr. C. A. G. Cooke, son of Mr. Arthur Cooke, M.B., Ch.B. Oxon., F.R.C.S., hon. assistant surgeon at Addenbrooke's Hospital, Cambridge, who was on board H.M.S. *Aboukir* as a midshipman when that vessel recently was torpedoed. He had been on duty until 5 A.M. that morning, and was in his bunk at the time. A terrific explosion occurred, and, thinking that his ship had struck a mine, as everyone else did, he rushed on deck. The ship listed considerably, but good order was maintained. Mr. Cooke made for the quarter-deck, where he found some of the officers and midshipmen. To the rails they hung as best they could, very soon realising that the ship was sinking. Then came the order "Every man for himself." Mr. Cooke jumped overboard, endeavouring to swim clear of the ship as fast as he could. Whilst in the water he saw H.M.S. *Hogue* and *Cressy* approaching, apparently about two or three miles apart and steaming towards the *Aboukir*. He made for the *Hogue*, and when within a distance of fifty yards that ship was also torpedoed twice, sinking in a few minutes. The *Hogue* was simply crumpled up in a most extraordinary manner. Mr. Cooke managed to catch hold of some of the wreckage, and was eventually picked up by one of the *Cressy's* cutters, after having been in the water about forty-five minutes. When picked up Mr. Cooke was clad in his pyjamas, and was transferred to the *Coriander*, a fishing trawler from Lowestoft, which in the ordinary way carries a crew of five, but on this occasion had about ninety survivors, having neither food nor clothes. They were on board the *Coriander* in this condition, with the sea continually washing over. About noon these survivors were taken on board the destroyer *Legion*, arriving at Harwich about 8.30 P.M.

Although he saw nothing of the submarines, Mr. Cooke is confident that one was sunk by the *Cressy*, several of the officers feeling equally confident that they saw the periscope of one shot away. The part of the *Aboukir* where Midshipman Cooke was on duty the previous night was shattered to pieces, hence his escape is almost miraculous. To some extent Midshipman Cooke owes his life to the fact that he is a good swimmer, and the reason so many of the officers were saved he attributes to the same fact. There was no distinction between officers and men, all had an equal chance. The *Aboukir* was torpedoed about 6.30 A.M. and sank about 7.5 A.M. The *Hogue* was struck at 7.10 A.M. and foundered about 7.15 A.M., and the *Cressy* was on the scene at 7.40 A.M.

